# GWAS of depression in 4,520 individuals from the Russian population highlights the role of *MAGI2* (*S-SCAM*) in the gut-brain axis

**DOI:** 10.3389/fgene.2022.972196

**Published:** 2023-01-04

**Authors:** Daria Pinakhina, Danat Yermakovich, Ekaterina Vergasova, Evgeny Kasyanov, Grigory Rukavishnikov, Valeriia Rezapova, Nikita Kolosov, Alexey Sergushichev, Iaroslav Popov, Elena Kovalenko, Anna Ilinskaya, Anna Kim, Nikolay Plotnikov, Valery Ilinsky, Nikholay Neznanov, Galina Mazo, Alexander Kibitov, Alexander Rakitko, Mykyta Artomov

**Affiliations:** ^1^ ITMO University, Saint-Petersburg, Russia; ^2^ Genotek Ltd., Moscow, Russia; ^3^ V.M. Bekhterev National Medical Research Center for Psychiatry and Neurology, Saint-Petersburg, Russia; ^4^ Almazov National Medical Research Center, Saint-Petersburg, Russia; ^5^ Broad Institute, Cambridge, MA, United States; ^6^ First Pavlov State Medical University of St. Petersburg, Saint-Petersburg, Russia; ^7^ Department of Pediatrics, The Ohio State University College of Medicine, Columbus, OH, United States; ^8^ The Institute for Genomic Medicine, Nationwide Children’s Hospital, Columbus, OH, United States; ^9^ Analytic and Translational Genetics Unit, Massachusetts General Hospital, Boston, MA, United States

**Keywords:** GWAS, depression, HADS-D, gene discovery, gut brain axis

## Abstract

We present the results of the depression Genome-wide association studies study performed on a cohort of Russian-descent individuals, which identified a novel association at chromosome 7q21 locus. Gene prioritization analysis based on already known depression risk genes indicated *MAGI2 (S-SCAM)* as the most probable gene from the locus and potential susceptibility gene for the disease. Brain and gut expression patterns were the main features highlighting functional relatedness of *MAGI2* to the previously known depression risk genes. Local genetic covariance analysis, analysis of gene expression, provided initial suggestive evidence of hospital anxiety and depression scale and diagnostic and statistical manual of mental disorders scales having a different relationship with gut-brain axis disturbance. It should be noted, that while several independent methods successfully *in silico* validate the role of *MAGI2*, we were unable to replicate genetic association for the leading variant in the *MAGI2* locus, therefore the role of rs521851 in depression should be interpreted with caution.

## Introduction

Globally around 1-in-7 people have one or more mental or substance use disorders, which results in mental health problems becoming one of the leading causes of disability worldwide resulting in the decrease in expected lifespan by up to two decades (Laursen, 2011; Mitchell, 2019; Dattani et al., 2022).

Depression is one of the most common mental health disorders. Several psychometric scales are used for screening and evaluation of the severity of depression. Importantly, such scales assess different symptomatics of depression. For example, the Hospital Anxiety and Depression Scale (HADS) focuses only on non-physical symptoms and is tuned for detection of depressive status at the time of the survey (Brennan et al., 2010). On the contrary, the Diagnostic and Statistical Manual of mental disorders, fifth edition (DSM-5) depression criteria include a considerable number of physical symptoms.

The clinical presentation of depression varies among patients, but could be clustered into distinct subphenotypes, representing a range of biochemical mechanisms (Belujon and Grace, 2017). The heterogeneity in ascertainment criteria and diagnostic instruments result in study cohorts consisting of depression subphenotypes with varying underlying genetic architecture (Howard et al., 2018; Cai et al., 2020a; Nguyen et al., 2021). However, the relationship between genetic architecture of subphenotypes and commonly used diagnostic scales are still underexplored.

Genome-wide association studies (GWAS) were successfully used over the years to study the genetic basis of diseases at a population scale, and in particular-depression. For example, recent meta-analyses discovered 102 independent variants and 269 genes associated with this condition (Howard et al., 2019). Phenotyping of depression cohorts for GWAS is usually performed with mood disorders questionnaires, a DSM-5 code in the medical history, or a semi-structured interview based on the DSM-5 diagnostic criteria. However, DSM does not reflect the whole range of genetic heterogeneity of depression, which is hypothesized to be one of the reasons for challenges in replication of depression GWAS findings (Adam, 2013; Marshall, 2020). A range of GWAS studies using other phenotyping approaches for depression, including symptom-based ones, were performed (Hek et al., 2013; Okbay et al., 2016). Several studies considered the HADS-D scale, implying that a more homogenous depression phenotype detected by this scale might increase the power of GWAS studies (Laurin et al., 2015; Taporoski et al., 2015; Heinzman et al., 2019).

The need for the development of a more holistic approach to diagnose mental health conditions, considering not only neuropsychiatric traits in this process, is indicated by the observation of significant comorbidities of psychiatric diseases with diseases of the gastrointestinal tract, cardiovascular system, and immunity (Steffen et al., 2020). For example, Mendelian randomization studies indicated a significant relationship between depression and gastrointestinal conditions, such as irritable bowel syndrome (IBS) and inflammatory bowel disease (IBD) (Wu et al., 2021a). It is known that the sole modulation of the gut microbiome can alleviate symptoms of depression in certain patients (Peirce and Alviña, 2019). Therefore, understanding of depression comorbidities and their genetic basis could aid in development of a better classification of depression subtypes and detection of individuals that would benefit from a particular type of treatment.

Here, we present a depression GWAS study of a cohort of Russian descent individuals identifying *MAGI2* as a potential susceptibility gene that could link inherited risks of depression and gastrointestinal conditions, associated with changes in gut permeability. We provide genetic analysis for different diagnostic criteria, suggesting potential utility of the HADS scale in genetic studies of depression. While the sample size of the cohort is modest, it is unique in the number of samples representing a previously under-reported in genetic studies Russian population (Kolosov et al., 2022).

## Materials and methods

### Data collection

The study was approved by an independent ethical committee in St. Petersburg Bekhterev Psychoneurological Research Institute (protocol #7 from 22.06.2017) and by Genotek Ltd., ethics committee (protocol #12, from 26.10.2019), all procedures were performed in accordance with the World Medical Association Declaration of Helsinki.

The cohort included male and female respondents over 18 years old, previously not ascertained for psychiatric disease status, who voluntarily provided their personal data and signed an electronic informed consent. Personal information was anonymized in the study. Participants were recruited from all around Russia, primarily representing larger metro areas. All participants provided a 2 ml sample of saliva that was used for DNA extraction and genotyping. DNA extraction was performed with QIAamp DNA Mini Kit (Qiagen). Biospecimens were obtained through at-home self-collection and mailing the sample or visiting the Genotek Ltd., laboratory (Moscow, Russia).

The phenotypic questionnaire was provided to participants through Genotek Ltd., website and consisted of three blocks: 1) questions about sex and age; 2) HADS online version—consisting of two independent subscales for anxiety (HADS-A) and depression (HADS-D); 3) DSM-5 based questions. Russian language adaptations of the questionnaires were used (Supplementary Materials, Cohort phenotyping, Supplementary Table S1) (Andriushchenko et al., 2003; Kasyanov et al., 2022). In total 6,178 individuals participated in the study.

### Genetic and phenotypic data quality filtration

The genotyping was performed using the Illumina Infinium Global Screening Array v2 (GSA). Genetic data was subjected to quality filtering. We eliminated samples with genetic and reported sex mismatch, low call rate (<.98), abnormal heterozygosity (>3 standard deviations, based on autosomal LD-pruned variants).

Only good quality DNA variants were kept for analysis using the Hardy-Weinberg equilibrium (pHWE > 1 × 10^−5^), call rate (>.98), and minor allele frequency (MAF > .01) filters. GWAS was performed only for variants with MAF > .05 to ensure sufficient statistical power.

Genotype imputation was performed using Haplotype Reference Consortium (HRC) and 1000 Genomes reference panels using Beagle 5.1 (Auton et al., 2015; McCarthy et al., 2016; Browning et al., 2018; Browning et al., 2021). Imputed variants with dosage R-squared DR2 > .7 were kept for the downstream analysis (Browning and Browning, 2007; Auton et al., 2015). Principal component analysis was performed with plink 2.0 (Chang et al., 2015).

Height and weight questions were used to identify likely non-reliable self-reported questionnaire responses—individuals outside of ranges 140–220 cm and/or 40–150 kg were excluded from the study (Supplementary Figure S1). Additionally, respondents who did not complete the entire questionnaire were excluded from the study. Of the remaining 5,795 participants, 5,724 completed the HADS and DSM-5 questionnaires, six persons did not report the gender, and were excluded, resulting in 5,718 participants.

Relatedness within the cohort was assessed using PRIMUS and for pairs with >.2, only one of the samples was randomly selected. The final sample size was 4,520 individuals.

For all categorical phenotypes of depression, the dichotomic dissection was applied as “yes” for “cases” and “no” for “controls.” The main control group consisted of participants without depressive conditions, as indicated by DSM-based (major depressive disorder, bipolar disease, generalized anxiety disorder) or categorical HADS (HADS-D and HADS-A) scales. GWAS for each categorical phenotype was performed independently of each other. For the HADS-D GWAS, two models were used: linear model, which considers the score as a quantitative trait, and logistic model, which implies the qualitative nature of the phenotype (they are referred to as linear HADS-D and logistic HADS-D GWAS below). For HADS-D qualitative phenotype, the subjects with ≥8 points were considered as cases and <8 points – as controls. In the DSM-5 criteria-based GWAS, a logistic model was applied.

### Variant-to-gene mapping and gene prioritization

The variants from the GWAS meta-analysis summary statistics on depression performed by Howard et al. (2019), from the GWAS on ICD-coded MDD phenotype reported by Howard et al. (2018), and from the GWAS on the lifetime MDD phenotype from Cai et al. (2020b), which have surpassed the threshold *p*-values of 5 × 10^−8^, 1 × 10^−6^, and 5 × 10^−5^ respectively, along with the variants associated with quantitative HADS-D scale in our data with *p*-value < 5 × 10^−6^ were merged into a single dataset and mapped to corresponding genes using POSTGAP (Ensembl Project, 2021). The GPrior (Kolosov et al., 2021) preprocessing module was then used to summarize the POSTGAP results to acquire gene-level features. Next, each gene was annotated with expression values in brain cell types and regions using data from Dropviz (Saunders et al., 2018), NeuroExpresso (Mancarci et al., 2017) and Allen Brain Atlas (Hawrylycz et al., 2012). The latter annotation was performed using the R package ABAData (Grote. ABAData, 2020). BiomaRT (Smedley et al., 2015) was used to convert mouse gene symbols to human. True and validation sets of genes for GPrior were generated from the genes, which were reported as significantly associated with depression in the GWAS Catalog (Buniello et al., 2019). Feature importance analysis for prioritization was performed using the R package randomForest (Ishwaran and Kogalur, 2021).

### Enrichment and semantic similarity analyses

The R package fgsea (Korotkevich et al., 2019) was used to estimate enrichment score of the IBS sigmoid colon differential expression data from the study by Videlock et al. (2018) with the genes associated with the linear HADS-D scale. The KEGG pathway enrichment analysis was performed using the clusterProfiler (Wu et al., 2021b) R package.

The genes, associated with the variants that achieved *p*-values of 5 × 10^−6^ in depression GWAS based on the DSM, and the HADS (linear and logistic models) scales, along with the genes from the set of differential expression analysis in IBS Videlock et al. (2018), were annotated with GO terms using the data from the ontology Similarity package (Greene et al., 2017). The terms were filtered by the information content (the threshold of .5 was used, the data is supplied with the package). Based on the obtained GO (Ashburner et al., 2000; Gene Ontology Consortium, 2021) annotations, frequency of the terms associated with each gene was computed [the package stopwords (Benoit et al., 2022) and an additional custom list of stop words were used for filtering the words before the computation]. FactoMineR (Lê et al., 2008) library was used to perform the PCA based on the frequencies of the terms associated with depression scales (HADS and DSM), and IBS transcriptomic data. The factoextra (Kassambara and Mundt, 2020) package was used to visualize the results. K-means function from the stats R package was used to cluster the terms based on their PCA coordinates. Finally, the GOSemSim (Yu, 2020) R package was used to perform a semantic similarity estimation between the sets of genes, associated with each scale-model combination considered, and the IBS gene set.

### Local genetic covariance and polygenic risk scores

We used the results of the study by Liu et al. (2015) (GWAS ID ieu-a-294) to investigate the association between depression and IBD using the analysis of local genetic covariance (Pierce and Burgess, 2013; Davey Smith and Hemani, 2014; Zhang et al., 2021). The packages TwoSampleMR and MR Egger for data pre-processing (Burgess et al., 2013; Bowden et al., 2015; Hemani et al., 2018).

We also calculated the polygenic risk scores (PRS) for the study cohort for IBD and depression, using summary statistics from Khera et al. (2018) and Coleman et al. (2020) (PGS Catalog ID: PGS000017, PGS000193, respectively). PRSice-2 was used to calculate the PRS (Choi and O’Reilly, 2019).

## Results

**FIGURE 1:**
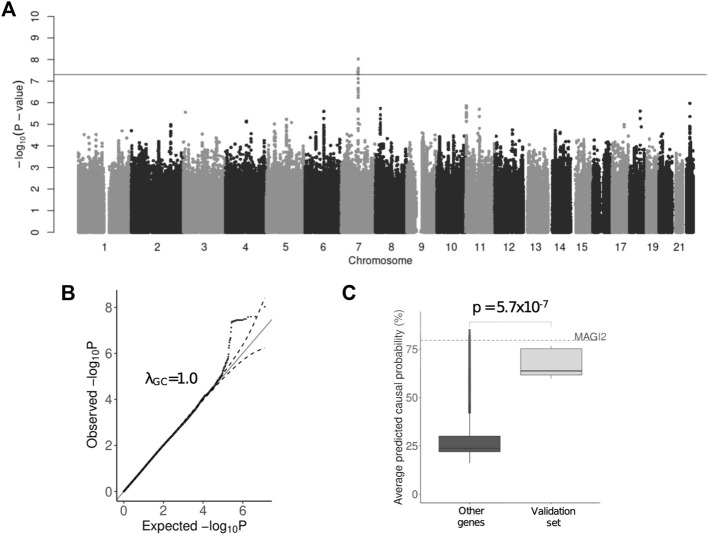
Results of the HADS-D-based GWAS. **(A)** Genome-wide significant signal on chromosome 7—rs521851; **(B)** QQ-plot showing test statistic; **(C)** The results of gene prioritization using GPrior.

Initially, we performed GWAS using the HADS-D subscale points (quantitative phenotype) with linear regression model corrected for age, sex and first ten principal components (Supplementary Figure S2). A significant association with 24 genome-wide significant variants (*p* < 5 × 10^−8^) was observed in 7q21 locus with the leading variant - rs521851 (*p* = 9.47 × 10^−9^, beta = .54). There were overall 40 variants with *p* < 10^−6^ and no inflation of test statistic was observed (Figures 1A, B). Using HapMap3 (Altshuler et al., 2010) data for recombination hotspot positions in the CEU population, we defined a haplotype to which the leading variant belonged (Supplementary Figure S3A). The variants, located within the region, delineated by the outside SNPs with the *p* < 5 × 10^−4^ within the target locus (a total of 436 variants) were selected for validation (Supplementary Tables S2A–C).

We used summary statistics from a major depressive disorder (MDD) GWAS by Coleman et al. (2020) for validation. The replication was not observed for the leading variant in our dataset—rs521851 (*p* = .33 in trauma-unexposed and *p* = .24 in trauma exposed MDD in Coleman et al.). 45 variants that were in LD with the leading variant had *p*-value below nominal validation threshold (*p* < .05/436 = 1.1 × 10^−4^), however, none of these variants surpasses significant association threshold in our data.

We examined two variants - the leading variant in this locus in Coleman et al.—rs535355 (Coleman et al. *p* = 6.56 × 10^−7^; Russian cohort HADS-D: *p* = 5.01 × 10^−4^) and the most associated in our cohort variant that surpasses nominal validation threshold - rs491394 (Coleman et al., 2020, *p* = 2.02 × 10^−6^; Russian cohort linear HADS-D *p* = 1.57 × 10^−5^). Both variants were correlated with the leading variant in our GWAS (P/*R* ^2^/D' = < 1 × 10^−4^/0.112/0.768 for rs535355 and P/*R* ^2^/D' = < 1 × 10^−4^/0.179/1.0 for rs491394, Supplementary Figure S3A) and had the same directionality of effect in both studies. In addition, for these two variants we observed the same effect directionality for strictly defined depression phenotypes considered by Cai et al. (2020b), and its opposite effect for the no-MDD help-seeking condition (Supplementary Figure S3B). However, these observations do not constitute a formal replication of our findings.

Since the leading variant in the locus was imputed, we ruled out the possibility of an imputation artifact. The frequency of imputed variant was compared to a whole genome panel and no significant differences were observed, additionally a directly genotyped variant in this locus and rs12112897 (*p* = 8.95 × 10^−6^, beta = 0.426) demonstrated similar effect size to the imputed leading variant (Supplementary Materials, Imputation validation, Supplementary Table S3).

### Candidate gene mapping

Another significantly associated variant in the locus—rs73137258 was located in the 5′-untranslated region of the *MAGI2*, suggesting that association signal could be mapped to this gene. Variant-to-gene mapping performed with POSTGAP (Ensembl Project, 2021) indicated that all genome-wide significant variants in the associated locus were related to *MAGI2* (Supplementary Table S4). Expression patterns of *MAGI2*, based on the protein atlas data, are in line with its putative role in depression: the highest expression level for this gene is in the central nervous system (Supplementary Figure S4A) (Uhlén et al., 2015; MAGI2, 2021). Antibody staining levels of *MAGI2* protein across nervous system cell types and tissues also showed high expression of the gene in colon peripheral nerve/ganglion (Supplementary Figure S4B).

Candidate genes obtained from the POSTGAP mapping were further prioritized based on their functional relatedness to the known MDD risk genes using GPrior (Kolosov et al., 2021). We used 948 features (Supplementary Table S5) for gene annotation and trained the prioritization model based on the list of previously reported depression risk genes (Supplementary Table S6). A separate validation set of known genes has been used as a control for gene prioritization. *MAGI2* was ranked 11th out of 5,010 genes considered (Figure 1C). Altogether, both variant-level, gene-level and functional level evidence strongly suggest *MAGI2* as a novel risk gene for depression.

### Gene prioritization feature analyses and MAGI2 function

Through feature importance analysis for the set of the 948 gene-based features used in the gene prioritization with GPrior, we noted that apart from expression patterns in brain anatomical structures, expression in the intestine, particularly, in the sigmoid colon, was among the biological features with the highest mean decrease in Gini coefficient (Supplementary Figure S5). Interestingly, IBS and IBD, for which Mendelian randomization, along with other lines of evidence, suggested a significant comorbidity with MDD, are characterized by changes in expression patterns and composition of sigmoid colon mucus (Fond et al., 2014; Post et al., 2019; Luo et al., 2022). These changes in IBS with constipation (IBS-C) have been reported to indicate alterations in neuronal signaling (Videlock et al., 2018).

The importance of the sigmoid colon gene expression pattern for the prioritization of depression risk genes could therefore be related to the shared biochemical mechanisms involved in the development of depression and disturbances in gut-brain axis interactions. Consistently with these observations, among the types of nervous system cells, the staining of the *Magi-2* protein antibody reaches the highest levels in the peripheral nerves of the colon (Supplementary Figure S4B), according to the Human Protein Atlas (Uhlén et al., 2015).

### Functional relationship between depression, IBS and IBD

We found a significant (*p* = 4 × 10^−5^; Supplementary Figure S6A) overlap between the set of differentially expressed genes in sigmoid colon mucus among IBS-C patients and healthy controls reported by Videlock et al. (2018) with the set of the genes, obtained through POSTGAP mapping of the variants with P < 5 × 10^−6^ in the quantitative HADS-D GWAS in the Russian cohort. The overlap between these sets is significantly higher than that of the quantitative HADS-associated set with random sets of genes with the same expression profile in the sigmoid colon as the IBS-C set (*p* = 4 × 10^−5^; Supplementary Figure S6A). Furthermore, there was a significant enrichment of differentially expressed IBS-C genes with the aforementioned set of quantitative HADS-D-associated genes (*p* = 0.014; Supplementary Figure S6B).

This set of genes obtained from POSTGAP mapping also demonstrates a significant enrichment with cAMP signaling pathway (*p* = 2.17 × 10^−5^, *p*.adj. = .005) (Supplementary Figures S6C, D) whereas weighted gene coexpression network analysis performed by Videlock et al. (2018), has revealed that cAMP/PKA signaling pathway is implicated in IBS-C. These results indicate that there could be a common genetic architecture involved in both the IBS-C and phenotype measured by HADS-D points. The genes associated with the linear HADS-D scale identified here are also enriched in the Alzheimer disease (AD) KEGG gene set (*p* = 1.78 × 10^−4^, *p*.adj. = .012). The risk of neurodegenerative diseases, including AD, has been shown to increase in IBD patients, and preclinical models based on AD also indicated aggravation of AD in case of comorbidity with IBD (Kim et al., 2022; Wang et al., 2022). Finally, we investigated whether other depression scales, such as DSM-5 or qualitative HADS-D could detect similar gut-brain axis interactions. We performed the same analysis for the qualitative HADS-D and DSM-based GWAS summary statistics obtained on the same cohort (Supplementary Figure S7). For qualitative HADS, the overlap with the IBS-C sigmoid colon mucus gene set is not significant, but appears to be retained at a tendency level, (*p* = .099, empirical), while for the DSM scale it is not traced at all (*p* = .39, empirical) (Supplementary Figure S8). The results of the GO annotation-based principal component analysis (PCA) and semantic similarity analysis support the highest affinity of the IBS-C data with the quantitative HADS scale genetic signal (Supplementary Figure S9).

The differences in genetic relationships of depression identified with HADS-D and DSM scales with IBD were also observed in the results of local genetic covariance analysis. We found more loci with nominally significant covariance with IBD (at *p*-value thresholds of .01 and .05) for both HADS scale models (Supplementary Figure S10A). Among them were those with both positive and negative covariance, whereas for DSM all loci at these significance thresholds had negative covariance (Supplementary Figure S10). Based on the composition of these loci, both HADS scales formed a cluster separate from DSM (Supplementary Figure S10E).

Polygenic risk scores for IBD in our cohort were significantly associated with depression identified with HADS-D (*p* = 5 × 10^−4^, Supplementary Figures S11A, B). Similarly, an association was observed for IBD PRS with the PRS for depression identified with CIDI (PGS000017, *p* = 8.76 × 10^−5^, Supplementary Figures S11C, D). HADS-D depression formulation was also associated with PRS for depression based on CIDI from Coleman et al., 2020 (PGS ID PGS000193, *p* = 4.91 × 10^−3^, Supplementary Figures S11E, F).

## Discussion

Our study presents the first depression GWAS based on the cohort of Russian-descent individuals. The genome-wide association signal, identified in the study, was not replicated in other studies of larger scale. However, the lack of replication power is widely observed in depression GWAS and a range of reasons for this phenomenon have already been suggested. For example, subphenotypes of depression, identified with different phenotyping approaches, are reported to display non-overlapping true positive associations. The lack of replication is hypothesized due to the disparity in the genetic architectures of the subphenotypes (Howard et al., 2018; Cai et al., 2020a). Current study represents the largest GWAS on depression which used the HADS scale as a sole diagnostic criterion.

In addition, the extent to which etiological factors for depression differ across populations remains unknown. Variability in cultural norms around depression and study participation can incur ascertainment biases, while population-specific environmental factors (including the nature of stress exposures) can also contribute to heterogeneity of depression in different populations, which can result in replication difficulty. Investigating genetic architecture of neuro-psychiatric traits, and depression in particular, in different populations can thus enhance understanding of genetic heterogeneity of the phenotypes and enrich the knowledge on possible disease mechanisms.

Despite modest sample size, our study is unique in the number of samples of a previously under-reported in genetic studies, Russian population (Kolosov et al., 2022). There are very few GWAS studies that involve Russian-descent individuals, especially in genetics of neuro-psychiatric traits, with no samples listed as Russian-origin in the largest depression GWAS cohorts (Howard et al., 2018; Wray et al., 2018). The potential to find novel associations and difficulty of replication have thus been expected in the current study due to the novelty of the studied population and the phenotyping approach, which has rarely been used in previous studies. However, without replication it is impossible to assess robustness of presented genetic findings.

The novel candidate gene identified in the study—*MAGI2,* is an illustration of how genetic susceptibilities for depression could be associated with systemic symptoms related to psychiatric traits. The variants in *MAGI2* were previously associated with response to antidepressant treatment, as well as with hippocampal atrophy (Potkin et al., 2009). Notably, the latter is one of the most frequent structural neuroimaging features associated with MDD (Sapolsky, 2001; Opel et al., 2014; Santos et al., 2018). It has been shown that social stress, inducing depression-like behavior alters spine morphology in mice hippocampus, whereas *MAGI2* is involved in reduction of spine density during reductions of GluA2 expression after social defeat stress (Danielson et al., 2012; Iñiguez et al., 2016).

Expression patterns and functional profile of *MAGI2* suggest that it could be involved in the development of both depression phenotypes and gastrointestinal conditions, such as IBS and IBD. It belongs to membrane-associated guanylate kinase (MAGUK) proteins with inverted orientation. MAGUKs are synaptic scaffolding proteins, which play crucial role in spatial organization of presynaptic and postsynaptic compartments, mediate functioning of multiple G protein-coupled receptors (GPCRs), interacting with them through their PDZ domains (Hammad et al., 2016). *MAGI2* itself is considered as an essential gene associated with intestinal barrier function, being involved in tight junction assembly (González-Mariscal et al., 2003; Ma and Morel, 2022). Its impairment has been associated with ulcerative colitis, Crohn’s disease, and levels of antibodies involved in IBD (McGovern et al., 2009).

*MAGI2* is also interacting with a range of receptors for which multiple lines of evidence suggest involvement in development of both depression as well as of IBS and/or IBD symptoms. Among them are *VIPR1*, *HTR2A*, *CRHR1*, *ADRB1*. The ligands of the first receptor are pituitary adenylate cyclase activating peptide (PACAP) and vasoactive intestinal polypeptide (VIP). The PACAP system, in turn, is involved in the molecular pathways of the three main theories of depression (monoamine, neurotrophic, and endocrine), while VIP is shown to be aberrantly expressed in patients with IBS and has been hypothesized to be involved in its development (Albert et al., 2011; Del Valle-Pinero et al., 2015; Bednarska et al., 2017). Previous research in clinical practice showed that IBS was associated with higher levels of anxiety and depressive symptoms compared to the general population. Moreover, antidepressants were proved efficacious treatment for IBS symptoms (Ford et al., 2019; Hu et al., 2021).

In addition to the processes of the gut-brain axis, *MAGI2* has vital functions in the central nervous system. It is a unique synaptic scaffolding protein, localized both in excitatory and GABAergic synapses, where it may function as a site of multiprotein assembly and interaction, and regulate subsynaptic domain association. Among the known *MAGI2* binding proteins are neuroligin 1, neuroligin 2, NMDA receptors, GKAP, SynArfGEF, IgSF9b. It is involved in the coupling of IgSF9b to neuroligin 2 in the development of inhibitory synapses (Woo et al., 2013). Notably, IgSF9b has been one of the top findings in one of GWAS meta-analyses for MDD, even though its physiological role is unexplored (Shyn et al., 2011).

Abnormal *MAGI2* protein levels disrupt excitation/inhibition balance in neurons, and aberrant expression of *MAGI2* is shown to cause the loss of GABAergic synapses in hippocampal neurons (Shin et al., 2020). GABAergic transmission, in turn, is a vital factor controlling hippocampal neurogenesis and neural maturation, which are considered as cellular substrates of most antidepressant therapies. Even comparatively modest deficits in GABAergic transmission in GABAA-receptor-deficient mice were reported to be sufficient to result in cognitive, neuroanatomical, neuroendocrine, behavioral, and antidepressant drug response expected of an animal model of MDD. Subunit composition of GABAA receptors mediating GABA inhibition are known to accompany MDD (Luscher et al., 2011).

Thus, our results indicate that depression phenotypes measured by the HADS scale and DSM criteria might differ, and the HADS scale may capture the phenotype, in which gut-brain axis interactions might be involved to a substantial extent. The genes which are found to be associated with the linear HADS scale in the current study are enriched in Alzheimer disease KEGG gene set, which also shows connections with IBD [the risk of AD is increased in IBD patients (Kim et al., 2022; Wang et al., 2022)] and cAMP signaling pathway implicated in IBS-C based on transcriptomic evidence (Videlock et al., 2018). Whether there are common genetic mechanisms linking both Alzheimer disease and depression with IBD requires further studies.

*MAGI2*—the gene, linked to the identified association for quantitative HADS scale-based GWAS has diverse functions, engulfing both the support of intestinal homeostasis affected in IBD and IBS on the one hand, and development and stabilization of synapses (including GABAergic ones), HPA axis, and serotonin signaling regulation through interaction with *CRHR1* and *HTR2A*—the processes vital for MDD pathogenesis on the other hand. This indicates that *MAGI2* could be one of the genes involved in the processes driving comorbidity between depression and diseases of the gut-brain axis, such as IBS.

## Study limitations

The study has a range of limitations, among the major of which are modest sample size comprised of the clients of a private genotyping company and phenotyping based on an online self-reporting without direct medical examination, which, in turn, called for harsh height and weight filtration thresholds. These can be potential sources of bias, and the results obtained in the study still require further replication efforts and should be interpreted with caution.

## Data Availability

The user agreement (available at https://www.genotek.ru) states that disclosure of individual level genetic information and/or self-reported Information to third parties for research purposes will not occur without explicit consent. Due to the user agreement the individual level cannot be made directly available to scientific community but have to be accessed indirectly via Genotek Ltd. The GWAS summary statistics will be made available through Synapse storage.
