# Supplementary material for: GWAS of depression in 4,520 individuals from the Russian population highlights the role of MAGI2 (S-SCAM) in the gut-brain axis
Source: Front Genet. 2023 Jan 4;13:972196. doi: 10.3389/fgene.2022.972196 (PMC9845291; doi:10.3389/fgene.2022.972196)
Supplement: Supplementary file 1 [file DataSheet1.pdf]

GWAS of depression in 4,520 individuals from the Russian population highlights the role of *MAGI2 (S-SCAM)* in the gut-brain axis.

Daria Pinakhina[1], Danat Yermakovich[2], Ekaterina Vergasova[2], Evgeny Kasyanov[3], Grigory Rukavishnikov[3], Valeriia Rezapova[1,4,5], Nikita Kolosov[1,4,5], Alexey Sergushichev[1], Iaroslav Popov[2], Elena Kovalenko[2], Anna Ilinskaya[2], Anna Kim[2], Nikolay Plotnikov[2], Valery Ilinsky[2,3], Nicolay Neznanov[3,6], Galina Mazo[3], Alexander Kibitov[3], Alexander Rakitko[2,3] and Mykyta Artomov [5,7,8,9]

1 – ITMO University, Saint-Petersburg, Russian Federation

2 – Genotek Ltd., Moscow, Russian Federation

3 –V.M. Bekhterev National Medical Research Center for Psychiatry and Neurology, Saint-Petersburg, Russian Federation

4 – Almazov National Medical Research Center, Saint-Petersburg, Russian Federation

5 – Broad Institute, Cambridge, MA, USA

6 – First Pavlov State Medical University of St. Petersburg, Saint-Petersburg, Russian Federation

7 - Department of Pediatrics, The Ohio State University College of Medicine, Columbus, OH, USA

8 - The Institute for Genomic Medicine, Nationwide Children's Hospital, Columbus, OH, USA

9 - Analytic and Translational Genetics Unit, Massachusetts General Hospital, Boston, MA, USA

Correspondence: mykyta.artomov@nationwidechildrens.org

Competing interests are disclosed at the end of the manuscript

Cohort phenotyping 3
Genetic data processing, gene mapping and prioritization methods 4
Imputation validation 6
Figure S1. Distributions of height and weight in the cohort and filtration thresholds used in the study. 7
Figure S2. Principal component analysis. 8
Figure S3. *MAGI2* risk locus and the forestplots for the key variants with nominal replication. 9
Figure S4. MAGI2 expression across tissues and cell types based on the Human protein atlas. 10
Figure S5. Feature importance analysis for gene prioritization. 11
Figure S7. Results of qualitative trait HADS-D and DSM-D GWAS on the Russian cohort for common (MAF>0.05) SNPs. 13
Figure S8. Comparison between the genetic signature of IBS-C in sigmoid colon mucus and the sets of the genes associated with other depression phenotyping approaches considered in the current study. 14
Figure S9. Semantic similarity comparison between GO terms associated with the genes for different depression scales and IBS-C. 15
Figure S10. Local genetic covariance analyses between depression and IBD based on the HADS and DSM GWAS presented in the study and ieu-a-294 IBD (Liu et al., 2015) data. 16
Figure S11. Association between polygenic risk scores (PRS) for IBD from the study by Khera et al., 2018 (Polygenic Score (PGS) ID: PGS000017) and depression identified with HADS-D. 18
References 19

Cohort phenotyping

The data was collected from December 2017 to February 2020. Subjects with mood disorders symptoms were identified through self-reports through an online questionnaire and two phenotypic blocks were constructed. The study included respondents over 18 years old, both sexes, with a height between 140 and 220 cm and weight between 40 kg and 150 kg, who agreed to participate and provide their genetic information for the study.

1. **HADS-based phenotypes**. Categorical and quantitative phenotypes were based on the Hospital Anxiety and Depression Scale (HADS)[1]. The questionnaire had 7 questions, each of which was offered 4 options of answer, assessed from 0 to 3 points depending on the severity of the symptom.

HADS consists of two independent subscales for anxiety (HADS-A) and depression (HADS-D) that allow the separate analysis of these phenotypes. The quantitative assessment was performed according to the total scores of the depression subscale. The categorical assessment was performed with the categories with cut-off ≥ 8 points of total points for this subscale. It did not include questions related to autonomic and general somatic symptoms, mostly overlapping with other nosologies that may lead to false positive results. In addition, due to the simple structure, HADS is suitable for online use. The data accumulated over the decades of using HADS have shown that this scale is a reliable tool for screening anxiety and depression symptoms in general practice patients [2] .

2. **DSM-based phenotypes.** Alternative categorical phenotypes in our study were assessed with the original online self- questionnaire based on DSM-5 diagnostic criteria with funnel filters in the questions structure. We used diagnostic criteria for major depressive disorder (MDD) (lifetime and current phenotypes), bipolar disorder (BD) (lifetime and current depression phenotypes) and generalized anxiety disorder (GAD).

MDD and broad BD phenotypes
1 - Have you been depressed, low or sad for several weeks in a row? (no/yes)
IF YES, THEN 1.1. - Are you in this state now? (no/yes)
2 - Have you had a period (2 weeks or more) during which you got much less pleasure from what was enjoyable before? (no/yes)
IF YES, THEN 2.1. - Are you in this state now? (no/yes)
IF 1 and 2 ARE "YES", THEN
3 - During periods of depression or low mood, what symptoms did you experience?
(None of the mentioned / Appetite was better than usual / Appetite was worse than usual / Sleep problems / Slept longer than usual / Movement and speech were slower than usual / Fatigue or lack of energy / Feeling worthless or guilty)
4 - Have you ever had periods (a week or more) when you had an elevated mood, or were you so active and full of energy that you got into trouble? (no/yes)

The MDD group consisted of subjects who answered positively to questions №1 and №2 of the first block (subparagraphs 1.1 and 2.1 are not considered separately), and also selected at least 3 items in question №3. Subjects that marked "yes" for item №4 were excluded from the MDD group. Such subjects fell into the broad BD group. The GAD group consisted of subjects who answered positively to questions №1, №2, №3 of the second block, and also noted at least 3 items in question №4.

Russian language adaptations of HADS and DSM scales were used in the study, which were previously validated in the Russian population [3,4]. Tightening the criteria for subjects from the MDD group in the form of obligatory two positive answers to questions №1 and №2, which correspond to the two main symptoms of depression according to the DSM-5, was done deliberately for greater homogenity of the sample. In addition, to exclude subjects with probable bipolar disorder from this group, the main criterion for a manic episode was added, but without additional symptoms, which allows us to speak only of an extended phenotype of this disorder.

Control groups

The main control group consisted of participants without any DSM-based (MDD, BD, GAD) or categorical HADS (HADS-Depression and HADS-Anxiety) phenotypes.

Genetic data processing, gene mapping and prioritization methods

The R package fastman[5] was used to build the Manhattan plot for the GWAS results, and the QQ-plot was generated using a custom script. The associated haplotype, lying between the CEU population recombination hotspots identified from HapMap3 data, was visualized with LocusZoom 1.4[6]. The package LDlinkR[7,8] was used to calculate the values of r2 and D for variants within the locus based on CEU data (from the 1000 Genomes Project, 2015[9]). The package gwasforest[10] was used to generate the forest plots for the variants in the peak locus for comparison of their effect directionality and size in a range of depression GWAS studies.

The variants from the GWAS meta-analysis summary statistics on depression performed by Howard et al., 2019[11], from the GWAS on ICD-coded MDD phenotype reported by Howard et al. in 2018[12], and from the GWAS on the lifetime MDD phenotype from Cai et al., 2020[13], which have surpassed the threshold p-values of $5x10^{-8}$, $1x10^{-6}$ and $5x10^{-5}$ correspondingly along with the variants associated with linear HADS-D scale with p-value $<5x10^{-6}$ have been linked to corresponding genes using POSTGAP[14]. The GPrior[15] preprocessing module was then used to summarize the POSTGAP results to acquire gene-level features. Furthermore, each gene has been annotated with its expression values in a range of brain cell types and regions using data from Dropviz[16], NeuroExpresso[17] and Allen Brain Atlas[18]. The latter annotation was performed using the R package ABAData[19]. BiomaRT[20] was used to convert mouse gene symbols to human. True and validation sets of genes for GPrior were generated from the genes, which were reported as significantly associated with depression in the GWAS Catalog[21]. Feature importance analysis for prioritization was performed using the R package randomForest[22].

Data on consensus normalized *MAGI2* expression values in a range of tissues and antibody staining levels for its product between cell types were obtained from the human protein atlas [23–25].

The R package fgsea[26] was used to estimate enrichment score of the IBS sigmoid colon differential expression data from the study by Videlock et al.[27] with the genes associated with the linear HADS-D scale. The KEGG pathway enrichment analysis was performed using the clusterProfiler[28] R package.

The genes, associated with the variants that achieved p-values of $5x10^{-6}$ in depression GWAS based on the DSM scale (with the logistic model), and the HADS scale (with the linear and logistic models), along with the genes from the set of differential expression analysis in IBS mentioned above, were annotated with GO terms using the data from the ontologySimilarity package[29]. The terms were filtered by the GO IC supplied with this package (the threshold of 0.5 was used). Based on the obtained GO[30,31] annotations, frequency of the terms associated with each gene was computed (the package stopwords[32] and an additional custom list of stop words were used for filtering the words before the computation). Thus, frequencies of terms occurring in GO annotations of the corresponding genes were obtained for the DSM and HADS scales along with the IBS data. PCA was then performed to understand the differences between them based on the terms. The FactoMineR[33] library was used to perform the PCA and the factoextra43 package was used to visualize the results. Kmeans from the stats R package was used to cluster the terms based on their PCA coordinates. Finally, the GOSemSim[34] R package was used to perform a semantic similarity estimation between the sets of genes, associated with each scale model combination considered, and the IBS gene sets (the set of differentially expressed genes in the sigmoid colon in IBS and the set of genes from the cAMP WGCNA module from the study by Videlock et al.).

We used SUPERGNOVA to investigate local genetic covariance between IBD and depression using ieu-a-294 and depression data for HADS and DSM presented here. 145 approximately independent regions with common variants from 1000 Genomes project phase III (rare variants with MAF < 5% were filtered out), for which data were available in all studies, were used in the analysis. The regions were found among 2353 approximately independent regions generated with LDetect for European population provided with SUPERGNOVA[35]. Ggupset[36] was used to generate the plots comparing composition of covariated regions with ieu-a-294 in the depression GWAS. Hclust function (with default settings) from stats R package[37] was used to perform hierarchical clustering of the studied depression scales based on composition of nominally significant ($p<0.05$) covariated regions with IBD (ieu-a-294).

PRSice-2[38] was used to calculate PRS scores for IBD based on Khera, 2018[39] study (polygenic risk score (PGS) catalog ID PGS000017) and from Coleman et al., 2020[40] (PGS ID PGS000193). Glm function from stats R package was used to model the relationship between IBD PRS and HADS-D scores.

Imputation validation

To eliminate the possibility of imputation artifacts for the leading variant - rs521851, we examined directly genotyped variants in this locus and rs12112897 ($p=8.95x10^{-6}$, beta=0.426) demonstrated similar effect size to the imputed leading variant. Furthermore, we evaluated the accuracy of the imputation by comparing allele frequencies for rs521851 in the studied cohort and a whole genome sequencing panel of 107 individuals sampled from the Russian population (clients of Genotek Ltd. not ascertained for phenotypic status at the time of recruitment), and no statistically significant differences were found (AF depression cohort = 0.1088; AF whole-genome sequencing = 0.1038; gnomad NFE AF = 0.1000) (**Sup. Table S3**).

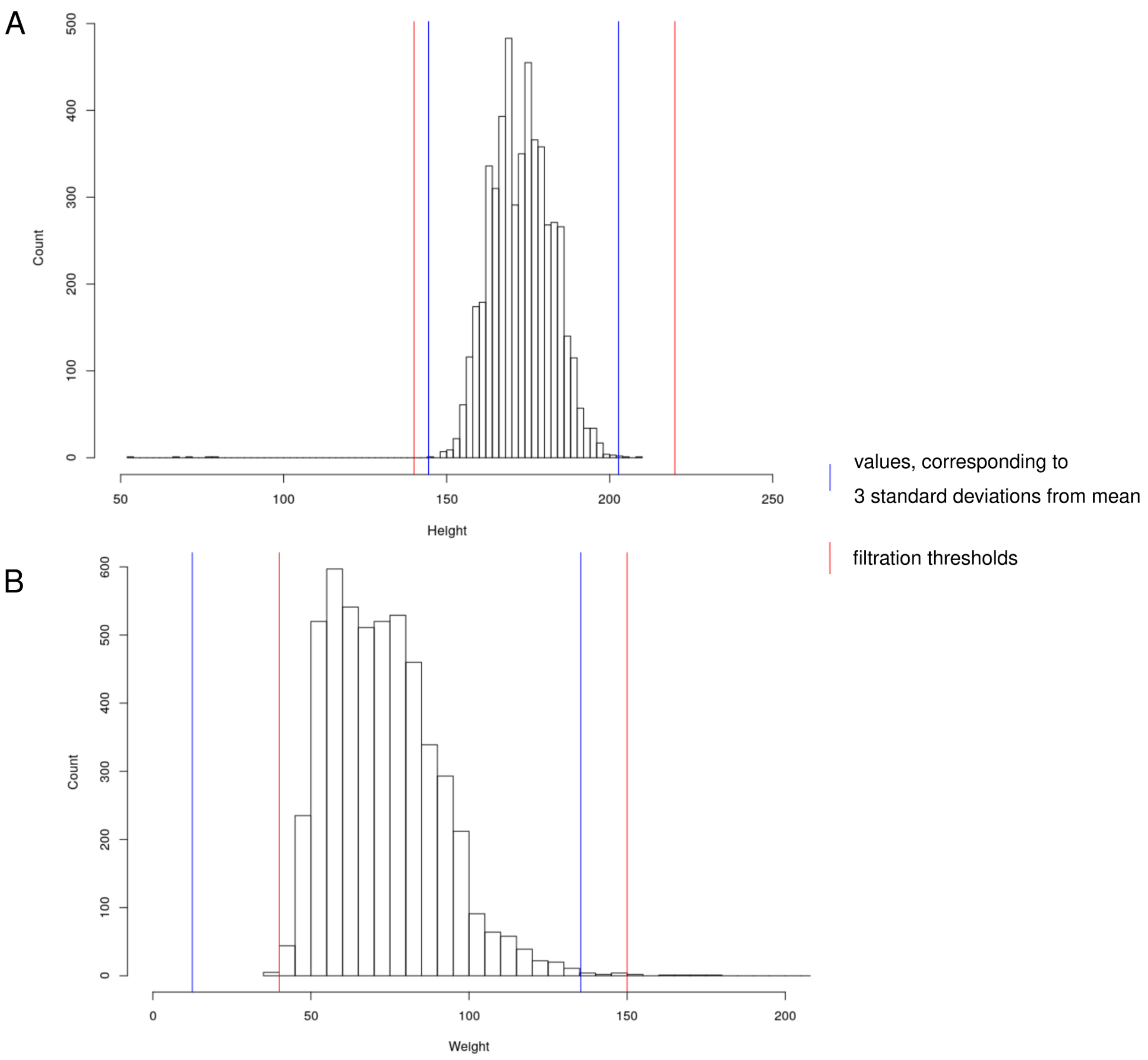

Figure S1. Distributions of height and weight in the cohort and filtration thresholds used in the study.

(A) Distribution of heights in the cohort, filtration thresholds used in the study (red), values, corresponding to 3 standard deviations from mean (blue); **(B)** Distribution of weights in the cohort, filtration thresholds used in the study (red), values, corresponding to 3 standard deviations from mean (blue).

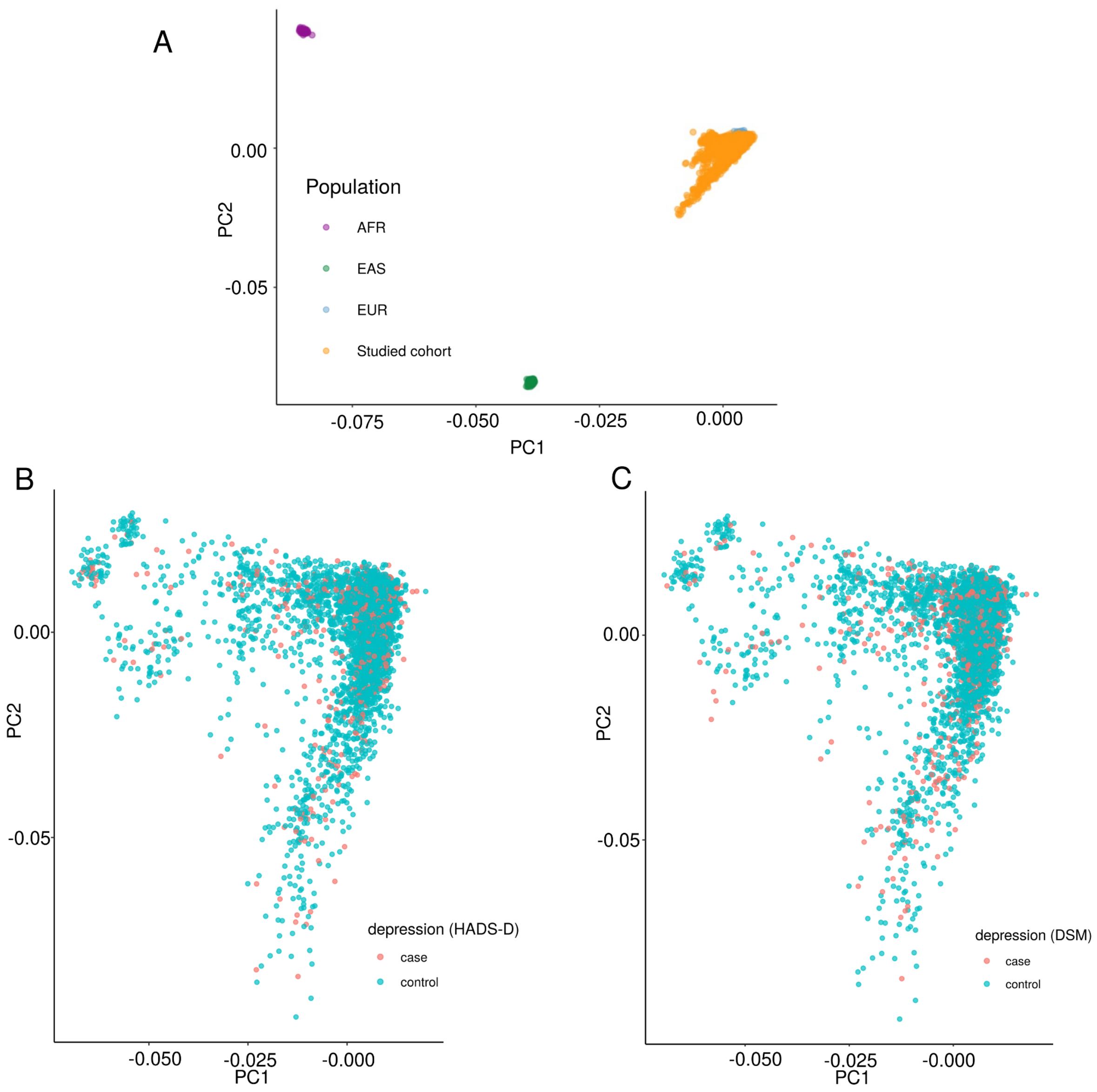

Figure S2. Principal component analysis.

(A) Study cohort PCA; **(B)** Depression cases labeled according to HADS-D depression diagnostic criterion; **(C)** Depression cases labeled according to DSM depression diagnostic criterion.

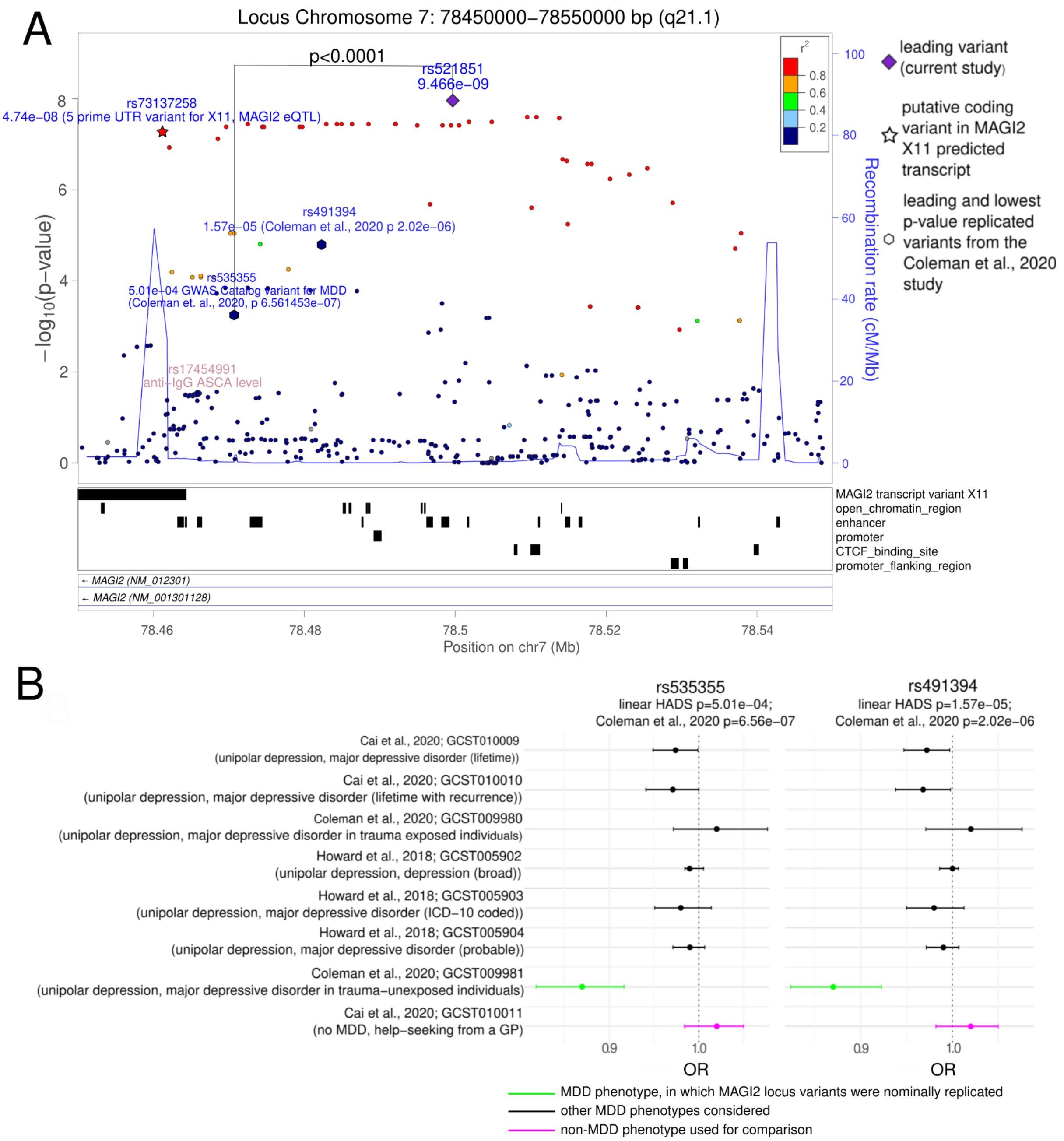

Figure S3. ***MAGI2*** **risk locus and the forestplots for the key variants with nominal replication.**

(A) The locus plot for the region with genome-wide significant SNPs on the chromosome 7 in the linear HADS-based GWAS on depression. The two SNPs with the smallest p-values in the locus; the leading and the lowest p-value variants from the Coleman et al., 2020 study along with the SNP correlated with IgA anti-Saccharomyces cervisiae antibody (ASCA) level as identified by McGovern et al., 2009[41] are marked. **(B)** The forest plots, comparing OR for two of the variants within the locus, replicated in the GWAS by Coleman et al, 2020: the leading variant in that study (rs535355) and the replicated variant with the lowest p-value in our study (rs491394).

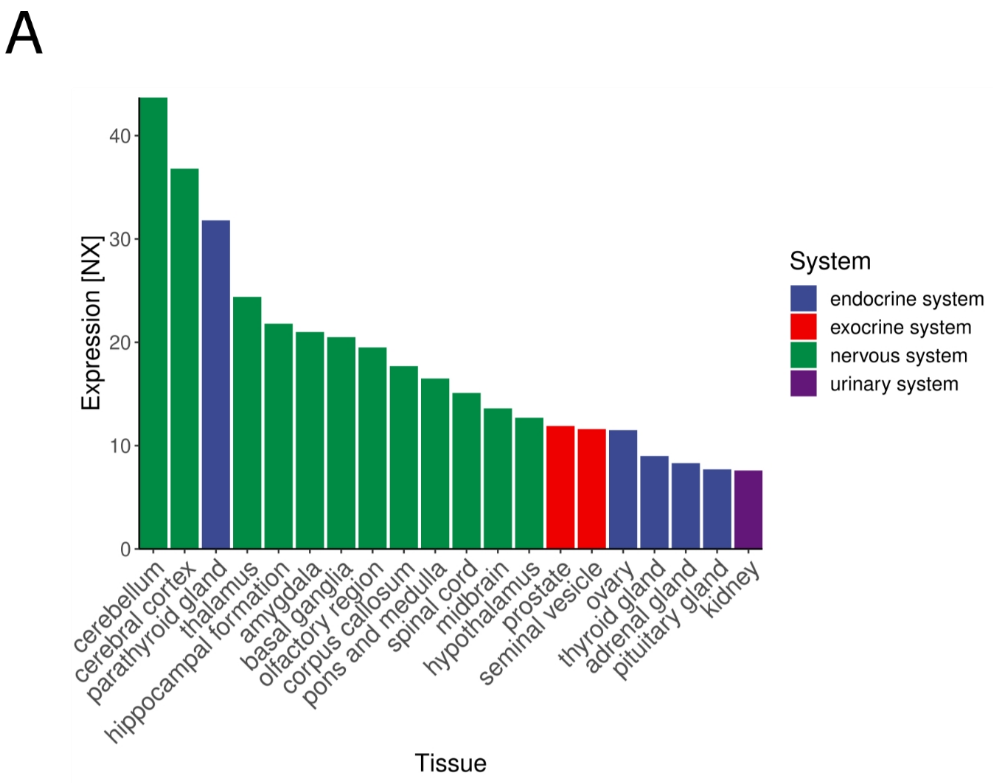

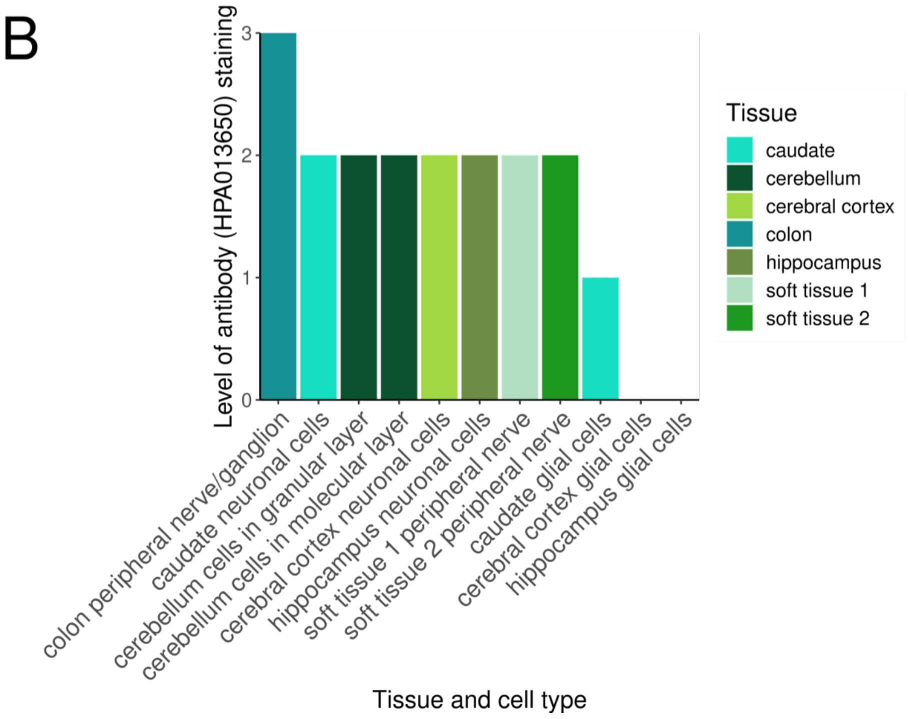

Figure S4. MAGI2 expression across tissues and cell types based on the Human protein atlas.

(A) Consensus normalized expression of MAGI2 across 20 tissues with the highest expression levels of this gene, obtained by combining the information from HPA, GTEx, and FANTOM5. (B) Antibody staining levels of MAGI2 protein across nervous system cell types and tissues (0 – not detected, 1 – low, 2 – medium, 3 – high).

A

B

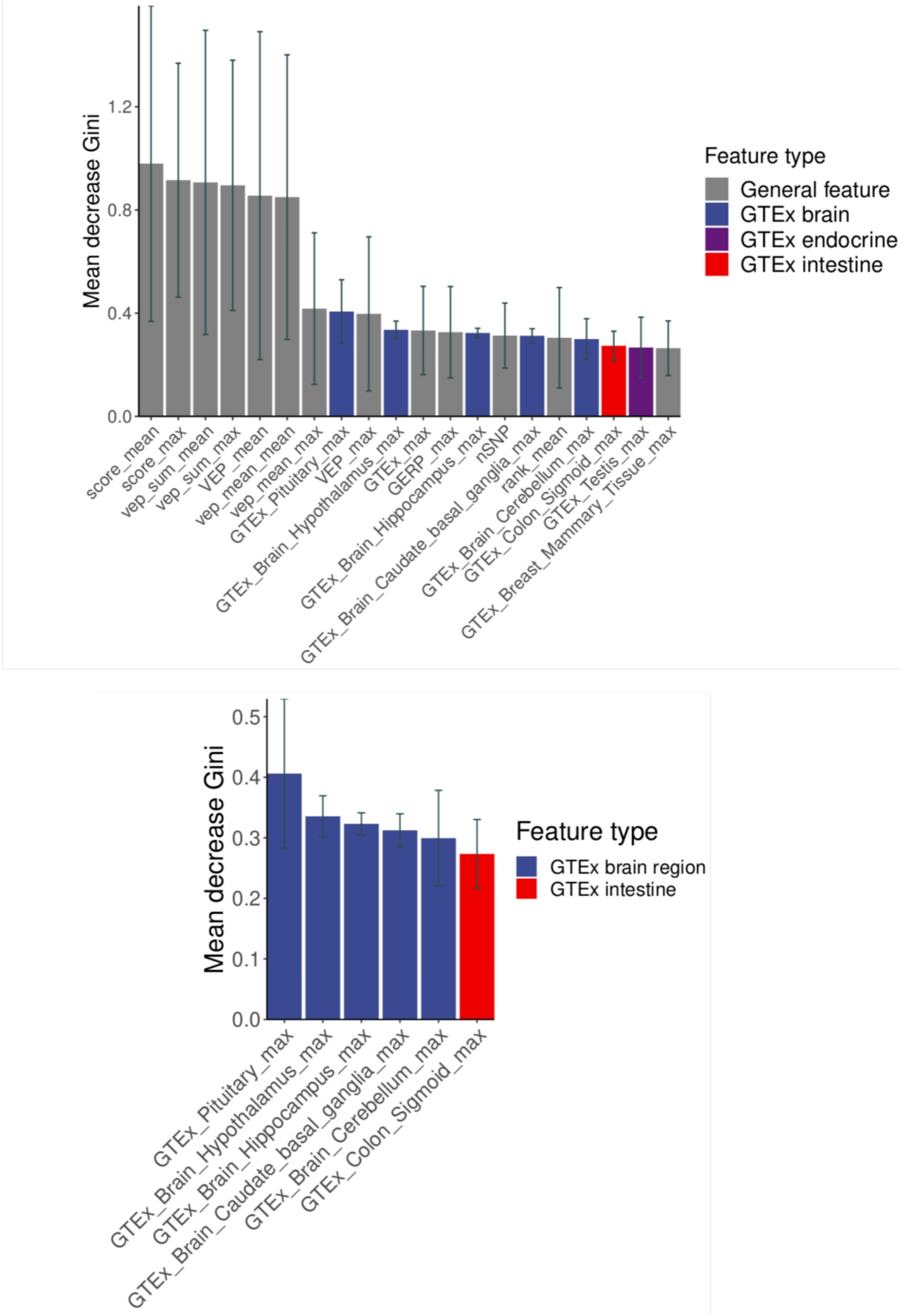

Figure S5. Feature importance analysis for gene prioritization.

(A) 20 features with the highest mean decrease Gini among the 948 features used in prioritization. (B) 6 GTEx features with the highest mean decrease Gini.

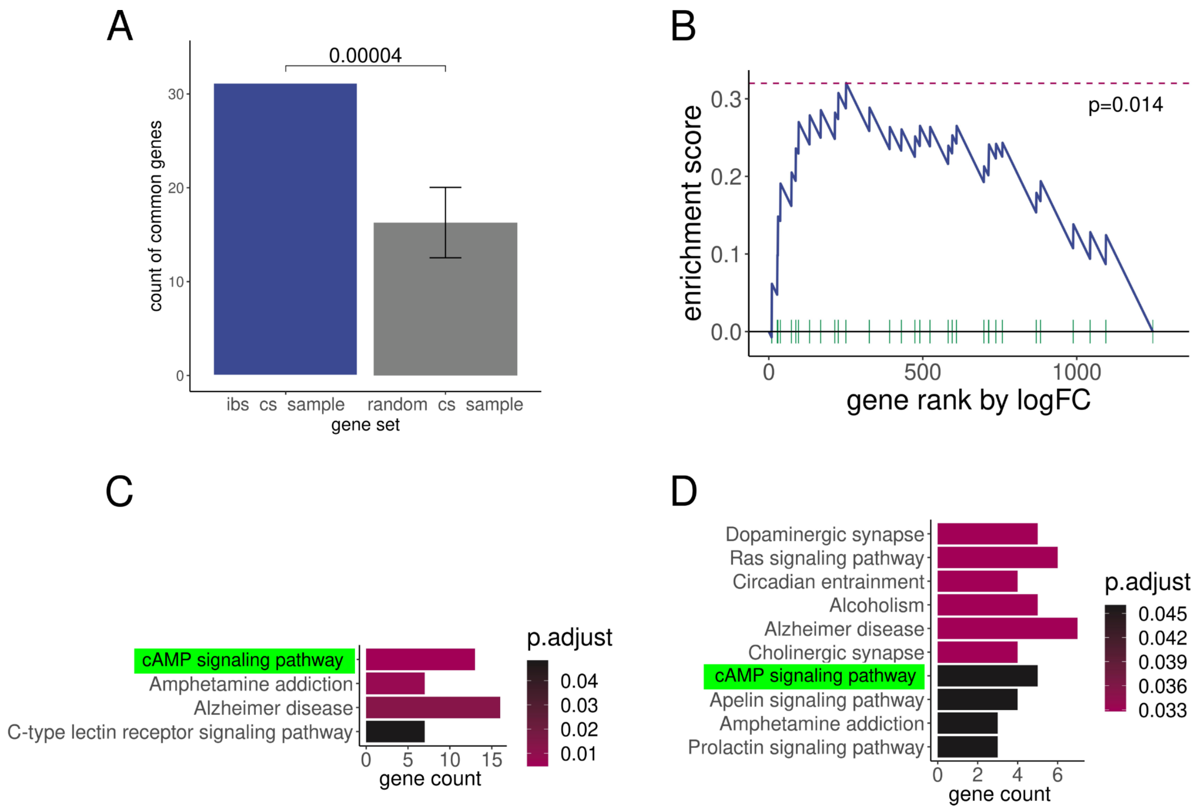

Figure S6. Comparison between the genetic signature of IBS-C in sigmoid colon mucus and the set of the genes associated with the linear HADS-D scale in current study.

(A) Difference in intersection sizes of the set of the genes, associated with the variants with p-value<5e-6 in the linear HADS-D GWAS with differentially expressed genes in sigmoid colon mucus of IBS-C patients compared with healthy controls (ibs cs sample; the data is from Videlock et al., 2018) and 100 random sets of genes with the same sigmoid colon expression profile (random cs sample; expression data is obtained from the GTEx portal*). (B) Enrichment plot, showing enrichment of the set of the genes from the aforementioned IBS-C differential expression set with the genes associated with the linear HADS-D scale estimated with fgsea. (C) KEGG enrichment of the set of the genes, associated with all variants with p-value<5e-6 in the linear HADS-based depression GWAS. (D) KEGG enrichment of the set of the genes, associated with linear HADS scale which are within the top 25% genes prioritized with GPrior.

*GTEx_Analysis_2017-06-05_v8_RNASeQCv1.1.9_gene_median_tpm.gct

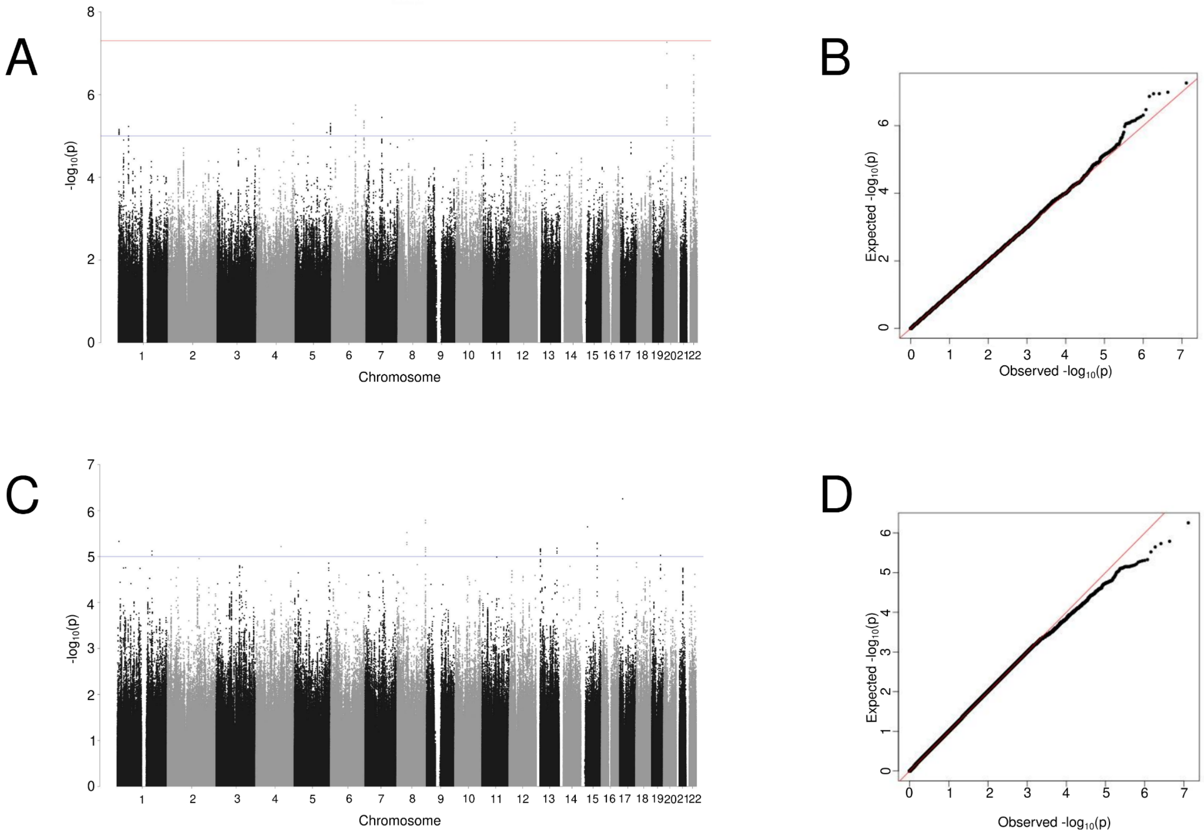

Figure S7. Results of qualitative trait HADS-D and DSM-D GWAS on the Russian cohort for common (MAF>0.05) SNPs.

(A) The Manhattan plot for the qualitative trait HADS-D study. (B) The QQ plot for the qualitative trait HADS-D study. (C) The Manhattan plot for the DSM-D study. (D) The QQ plot for the DSM-D study.

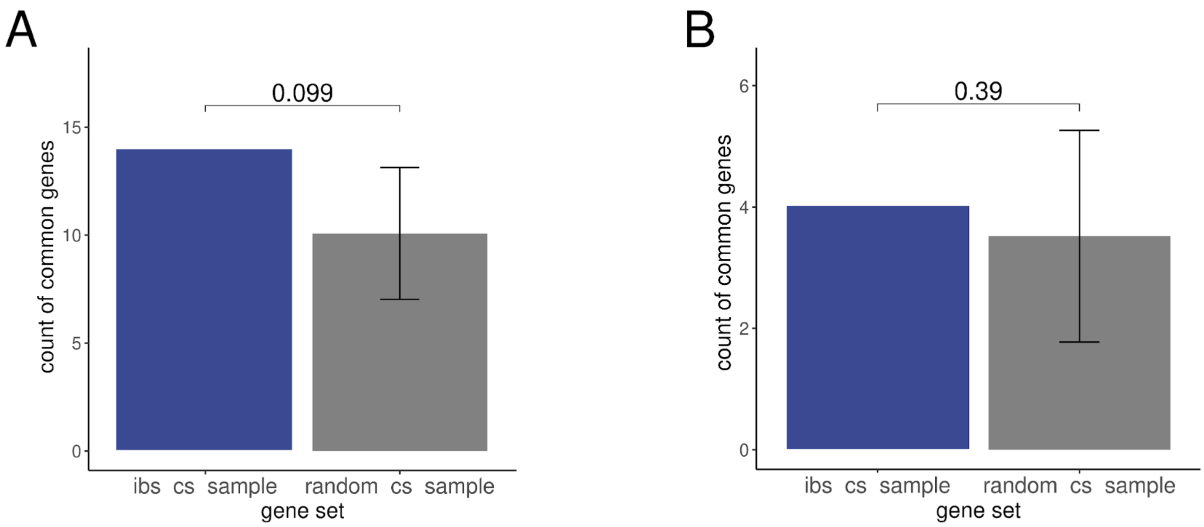

Figure S8. Comparison between the genetic signature of IBS-C in sigmoid colon mucus and the sets of the genes associated with other depression phenotyping approaches considered in the current study.

(A) Comparison between intersection sizes of the set of the genes, associated with the variants with p-value<5e-6 in logistic HADS-D GWAS with differentially expressed genes in sigmoid colon mucus of IBS-C patients compared with healthy controls (ibs cs sample; the data is from Videlock et al., 2018) and 100 random sets of genes with the same sigmoid colon expression profile (random cs sample; expression data is obtained from the GTEx portal*). (B) Comparison between intersection sizes of the set of the genes, associated with the variants with p-value<5e-6 in DSM GWAS with differentially expressed genes in sigmoid colon mucus of IBS-C patients compared with healthy controls (ibs cs sample; the data is from Videlock et al., 2018) and 100 random sets of genes with the same sigmoid colon expression profile (random cs sample; expression data is obtained from the GTEx portal*).
*GTEx_Analysis_2017-06-05_v8_RNASeQCv1.1.9_gene_median_tpm.gct

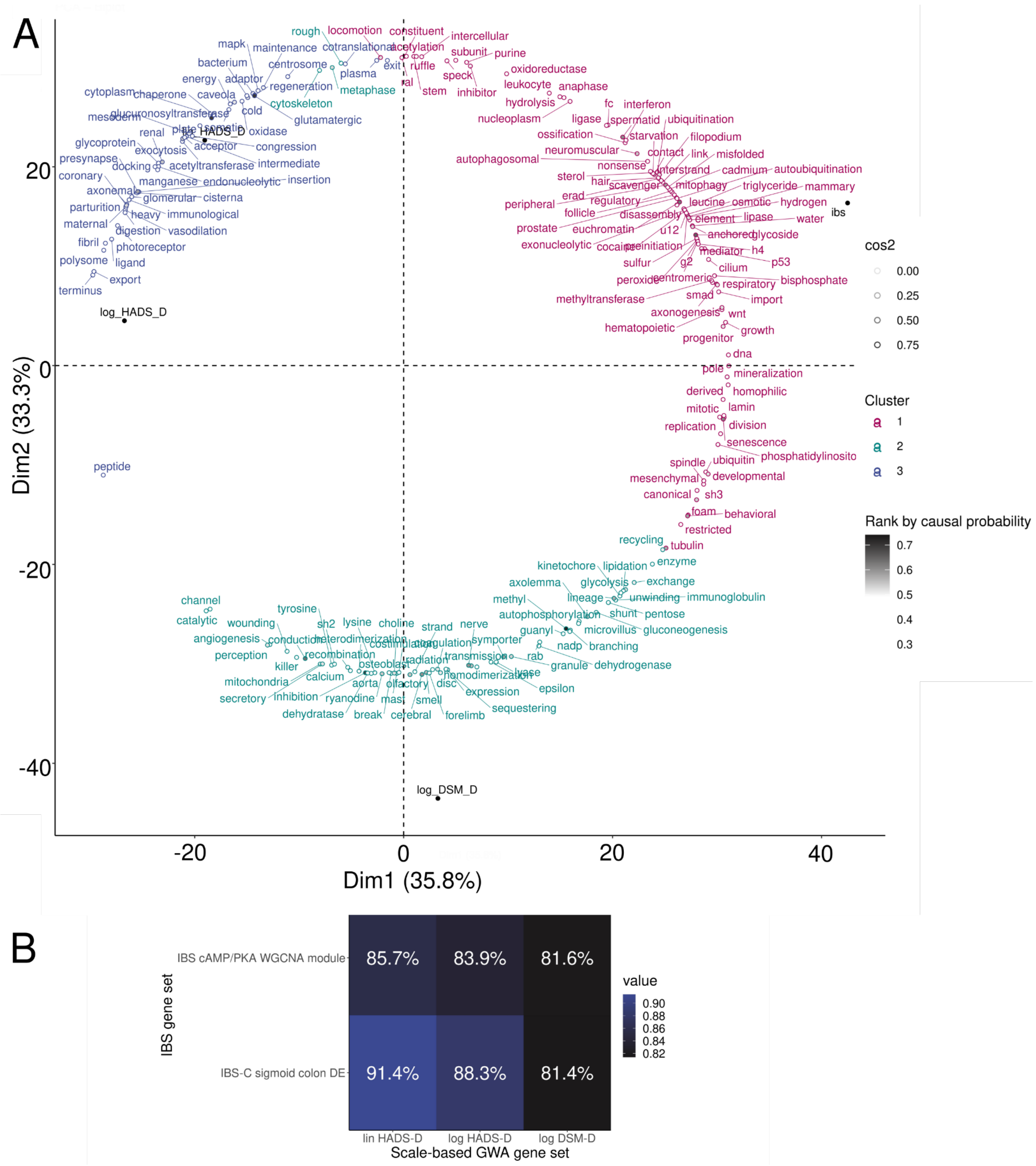

Figure S9. Semantic similarity comparison between GO terms associated with the genes for different depression scales and IBS-C.

(A) Biplot for the first two principal components (Dim1 and Dim2) showing the results of the PCA computed based on GO terms (BP, CC, MF) associated with the genes from the 4 sets: 15the 3 gene sets, associated with the variants with p-value <5e-6 in the GWAS studies on the scale types (logistic DSM-D, logistic HADS-D, linear HADS-D) and the genes differentially expressed in sigmoid colon of IBS-C patients (the data is from Videlock et al., 2018), which are shared with the genes used in gene prioritization for depression. The terms are organized into three clusters by k-means clustering and mean ranks are assigned to them based on Gprior probabilities of the genes with which they are associated. (B)

Semantic similarity between the sets of the genes, associated with each scale and model combination considered, and the IBS gene sets (the set of differentially expressed genes in the sigmoid colon in IBS and the set of genes from the cAMP WGCNA module from the study by Videlock et al.) estimated with GOSemSim.

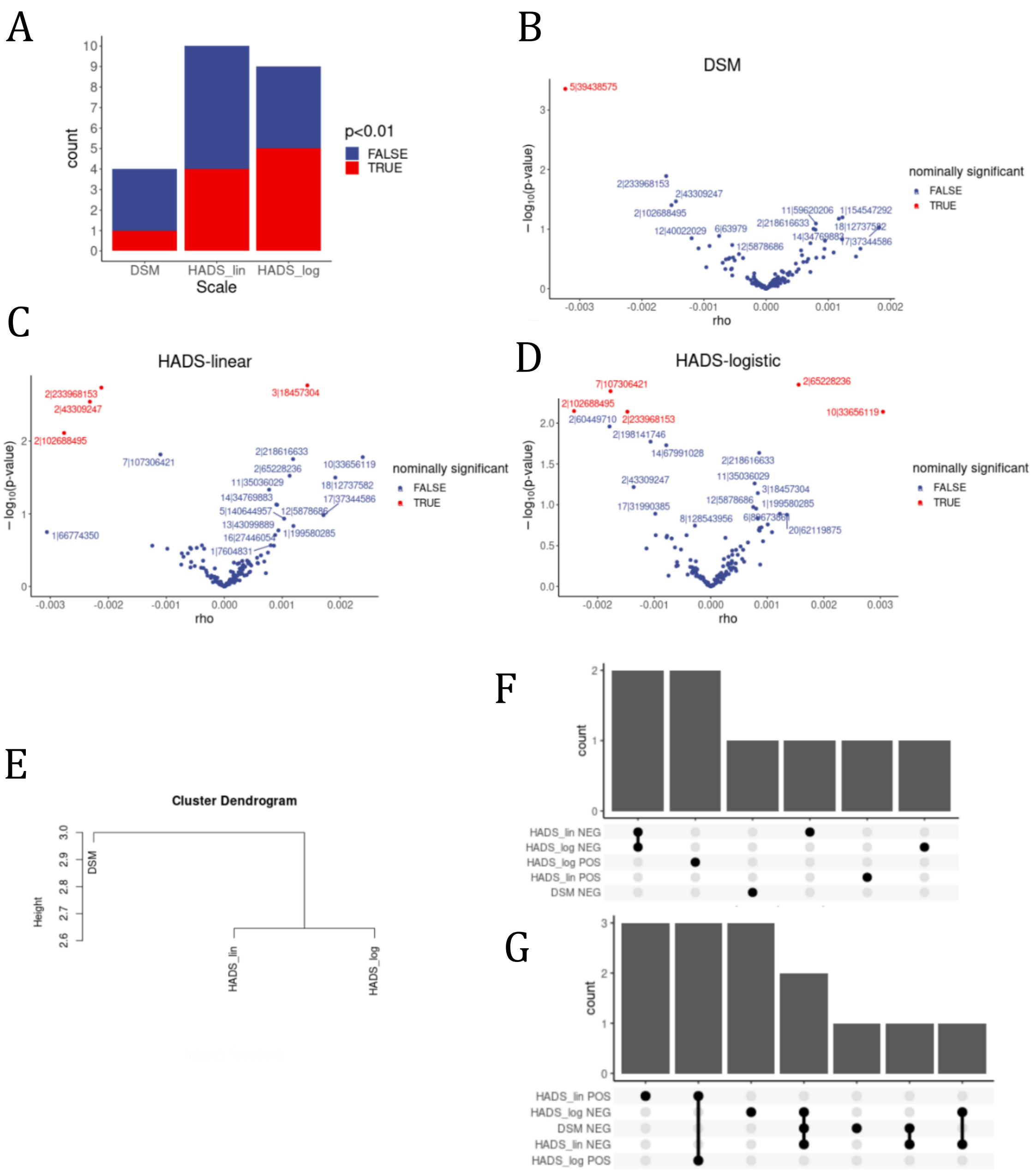

Figure S10. Local genetic covariance analyses between depression and IBD based on the HADS and DSM GWAS presented in the study and ieu-a-294 IBD (Liu et al., 2015) data.

(A) A bar plot showing counts of covariant regions between depression identified using HADS (qualitative-HADS_log, and quantitative-HADS-lin), and DSM, and IBD (ieu-a-294) that achieved nominal significance ($p<0.05$), the data for the regions with p-value < 0.01 is shown in red. (B) A scatterplot, showing the results of local genetic covariance analysis for the DSM data. Regions with p-value<0.01 are shown in red. (C) A scatterplot, showing the

results of local genetic covariance analysis for the quantitative HADS (HADS-linear) data. Regions with p-value<0.01 are shown in red. (D) A scatterplot, showing the results of local genetic covariance analysis for the quantitative HADS (HADS-linear) data. Regions with p-value<0.01 are shown in red. (E) A dendrogram, showing results of hierarchical clustering of DSM, qualitative and quantitative HADS (HADS_log and HADS_lin) data based on the composition of nominally significant (p<0.05) covariant regions with IBD (ieu-a-294). (F) An UpSet plot, comparing composition of regions with local genetic covariance with IBD (ieu-a-294) p-value<0.01 in HADS and DSM depression data. (G) An UpSet plot, comparing composition of regions with local genetic covariance with IBD (ieu-a-294) p-value<0.05 in HADS and DSM depression data.

A

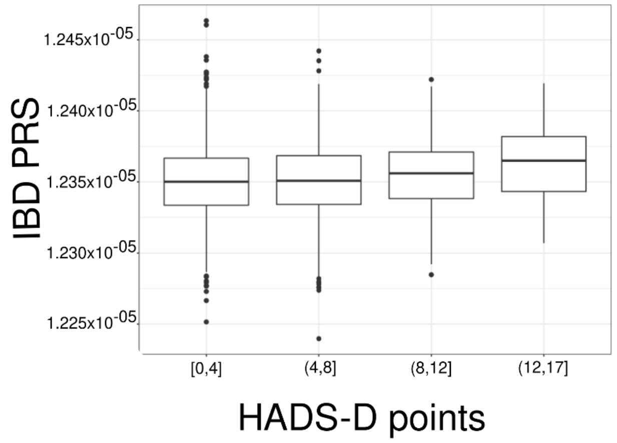

B

	Estimate	Std. Error	t-value	P-value
**Intercept**	-67.430	20.56	-3.280	0.001***
**IBD**	5820699.58	1664426.94	3.497	0.0005***

C

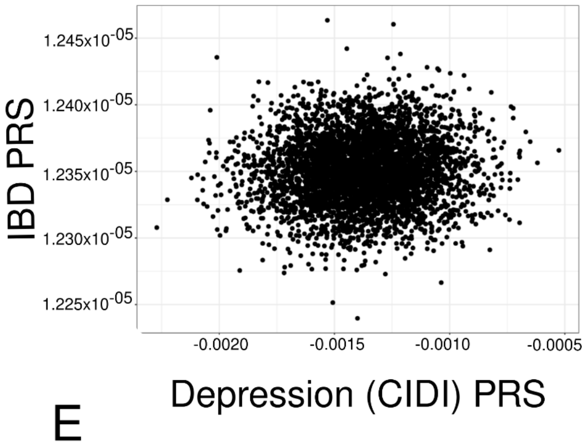

D

	Estimate	Std. Error	t-value	P-value
**Intercept**	-0.008	0.002	-4.751	$2.08x10^{-6}$***
**IBD**	529.82	134,949	3.926	$8.76x10^{-5}$***

E

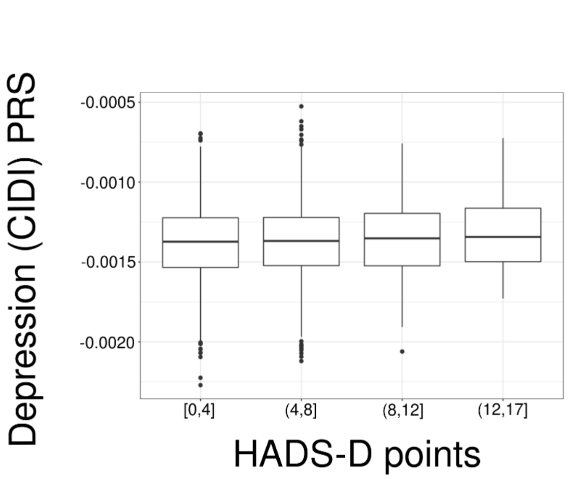

F

	Estimate	Std. Error	t-value	P-value
**Intercept**	5.167	0.256	20.218	$< 2x10^{-16}$***
**Depression (CIDI)**	515.711	183.269	2.814	0.00491**

Figure S11. Association between polygenic risk scores (PRS) for IBD from the study by Khera et al., 2018 (Polygenic Score (PGS) ID: PGS000017) and depression identified with HADS-D.

(A) Barplots, showing changes in HADS-D scores corresponding to those of IBD PRS (PGS000017). (B) Estimates of coefficients and their significance for a generalized linear model of HADS-D depression scores based on IBD PRS. (C) A scatterplot, showing the relationship between IBD (PGS000017) and depression (CIDI, PGS000193) PRS scores.

(D) Estimates of coefficients and their significance for a generalized linear model of relationships between IBD (PGS000017) and depression (CIDI, PGS000193) PRS. (E) Barplots, showing changes in HADS-D scores associated with depression (CIDI, PGS000193) PRS. (F) Estimates of coefficients and their significance for a generalized linear model of relationships between HADS-D scores and depression (CIDI, PGS000193) PRS.

References

1. Zigmond AS, Snaith RP. The hospital anxiety and depression scale. *Acta Psychiatr Scand*. 1983;67(6):361-370. doi:10.1111/j.1600-0447.1983.tb09716.x
2. Brennan C, Worrall-Davies A, McMillan D, Gilbody S, House A. The Hospital Anxiety and Depression Scale: a diagnostic meta-analysis of case-finding ability. *J Psychosom Res*. 2010;69(4):371-378. doi:10.1016/j.jpsychores.2010.04.006
3. Andriushchenko AV, Drobizhev MI, Dobrovol'skiĭ AV. [A comparative validation of the scale CES-D, BDI, and HADS(d) in diagnosis of depressive disorders in general practice]. *Zh Nevrol Psikhiatr Im S S Korsakova*. 2003;103(5):11-18.
4. Kasyanov ED, Verbitskaya EV, Rakitko AS, et al. [Validation of a DSM-5-based screening test using digital phenotyping in the Russian population]. *Zh Nevrol Psikhiatr Im S S Korsakova*. 2022;122(6. Vyp. 2):64-70. doi:10.17116/jnevro202212206264
5. danioreo/fastman: A Fast Way to Create Manhattan Plots for GWAS Data version 0.1.0 from GitHub. Accessed November 13, 2021. https://rdrr.io/github/danioreo/fastman/
6. Pruim RJ, Welch RP, Sanna S, et al. LocusZoom: regional visualization of genome-wide association scan results. *Bioinforma Oxf Engl*. 2010;26(18):2336-2337. doi:10.1093/bioinformatics/btq419
7. Myers TA, Chanock SJ, Machiela MJ. *LDlinkR: Calculating Linkage Disequilibrium (LD) in Human Population Groups of Interest*.; 2021. Accessed November 13, 2021. https://CRAN.R-project.org/package=LDlinkR
8. Machiela MJ, Chanock SJ. LDlink: a web-based application for exploring population-specific haplotype structure and linking correlated alleles of possible functional variants. *Bioinformatics*. 2015;31(21):3555-3557. doi:10.1093/bioinformatics/btv402
9. Auton A, Abecasis GR, Altshuler DM, et al. A global reference for human genetic variation. *Nature*. 2015;526(7571):68-74. doi:10.1038/nature15393
10. Xu Y, Wang A. *Gwasforest: Make Forest Plot with GWAS Data*.; 2020. Accessed November 13, 2021. https://CRAN.R-project.org/package=gwasforest
11. Howard DM, Adams MJ, Clarke TK, et al. Genome-wide meta-analysis of depression identifies 102 independent variants and highlights the importance of the prefrontal brain regions. *Nat Neurosci*. 2019;22(3):343-352. doi:10.1038/s41593-018-0326-7
12. Howard DM, Adams MJ, Shirali M, et al. Genome-wide association study of depression phenotypes in UK Biobank identifies variants in excitatory synaptic pathways. *Nat Commun*. 2018;9(1):1470. doi:10.1038/s41467-018-03819-3
13. Cai N, Revez JA, Adams MJ, et al. Minimal phenotyping yields genome-wide association signals of low specificity for major depression. *Nat Genet*. 2020;52(4):437-447. doi:10.1038/s41588-020-0594-5
14. *Post-GWAS Analysis Pipeline*. Ensembl Project; 2021. Accessed November 15, 2021. https://github.com/Ensembl/postgap
15. Kolosov N, Daly MJ, Artomov M. Prioritization of disease genes from GWAS using

ensemble-based positive-unlabeled learning. *Eur J Hum Genet EJHG*. 2021;29(10):1527-1535. doi:10.1038/s41431-021-00930-w
16. Saunders A, Macosko EZ, Wysoker A, et al. Molecular Diversity and Specializations among the Cells of the Adult Mouse Brain. *Cell*. 2018;174(4):1015-1030.e16. doi:10.1016/j.cell.2018.07.028
17. Mancarci BO, Toker L, Tripathy SJ, et al. Cross-Laboratory Analysis of Brain Cell Type Transcriptomes with Applications to Interpretation of Bulk Tissue Data. *eNeuro*. 2017;4(6). doi:10.1523/ENEURO.0212-17.2017
18. Hawrylycz MJ, Lein ES, Guillozet-Bongaarts AL, et al. An anatomically comprehensive atlas of the adult human brain transcriptome. *Nature*. 2012;489(7416):391-399. doi:10.1038/nature11405
19. Grote S, Prüfer K, Kelso J, Dannemann M. ABAEnrichment: an R package to test for gene set expression enrichment in the adult and developing human brain. *Bioinformatics*. 2016;32(20):3201-3203. doi:10.1093/bioinformatics/btw392
20. Smedley D, Haider S, Durinck S, et al. The BioMart community portal: an innovative alternative to large, centralized data repositories. *Nucleic Acids Res*. 2015;43(W1):W589-W598. doi:10.1093/nar/gkv350
21. Buniello A, MacArthur JAL, Cerezo M, et al. The NHGRI-EBI GWAS Catalog of published genome-wide association studies, targeted arrays and summary statistics 2019. *Nucleic Acids Res*. 2019;47(D1):D1005-D1012. doi:10.1093/nar/gky1120
22. Ishwaran H, Kogalur UB. *RandomForestSRC: Fast Unified Random Forests for Survival, Regression, and Classification (RF-SRC).*; 2021. Accessed November 13, 2021. https://CRAN.R-project.org/package=randomForestSRC
23. Uhlén M, Fagerberg L, Hallström BM, et al. Proteomics. Tissue-based map of the human proteome. *Science*. 2015;347(6220):1260419. doi:10.1126/science.1260419
24. MAGI2 protein expression summary - The Human Protein Atlas. Accessed November 13, 2021. https://www.proteinatlas.org/ENSG00000187391-MAGI2
25. Tissue expression of MAGI2 - Primary data - The Human Protein Atlas. Accessed November 13, 2021. https://www.proteinatlas.org/ENSG00000187391-MAGI2/tissue/primary+data
26. Korotkevich G, Sukhov V, Budin N, Shpak B, Artyomov M, Sergushichev A. Fast gene set enrichment analysis. Published online 2019. doi:10.1101/060012
27. Videlock EJ, Mahurkar-Joshi S, Hoffman JM, et al. Sigmoid colon mucosal gene expression supports alterations of neuronal signaling in irritable bowel syndrome with constipation. *Am J Physiol Gastrointest Liver Physiol*. 2018;315(1):G140-G157. doi:10.1152/ajpgi.00288.2017
28. Wu T, Hu E, Xu S, et al. clusterProfiler 4.0: A universal enrichment tool for interpreting omics data. *The Innovation*. 2021;2(3). doi:10.1016/j.xinn.2021.100141
29. Greene D, Richardson S, Turro E. ontologyX: a suite of R packages for working with ontological data. *Bioinformatics*. 2017;33(7):1104-1106. doi:10.1093/bioinformatics/btw763
30. Ashburner M, Ball CA, Blake JA, et al. Gene Ontology: tool for the unification of biology. *Nat Genet*. 2000;25(1):25-29. doi:10.1038/75556
31. Gene Ontology Consortium. The Gene Ontology resource: enriching a GOld mine. *Nucleic Acids Res*. 2021;49(D1):D325-D334. doi:10.1093/nar/gkaa1113
32. Benoit K, Muhr D, Watanabe K. *Stopwords: Multilingual Stopword Lists.*; 2021. Accessed November 13, 2021. https://CRAN.R-project.org/package=stopwords
33. Lê S, Josse J, Husson F. FactoMineR: An R Package for Multivariate Analysis. *J Stat Softw*. 2008;25:1-18. doi:10.18637/jss.v025.i01
34. Yu G. Gene Ontology Semantic Similarity Analysis Using GOSemSim. *Methods Mol Biol Clifton NJ*. 2020;2117:207-215. doi:10.1007/978-1-0716-0301-7_11
35. Zhang Y, Lu Q, Ye Y, et al. SUPERGNOVA: local genetic correlation analysis reveals heterogeneous etiologic sharing of complex traits. *Genome Biol*. 2021;22(1):262. doi:10.1186/s13059-021-02478-w
36. Ahlmann-Eltze C. *Ggupset: Combination Matrix Axis for "ggplot2" to Create "UpSet"*

Plots.; 2020. Accessed September 16, 2022. https://CRAN.R-project.org/package=ggupset
37. R Core Team. *R: A Language and Environment for Statistical Computing*.; 2020.
38. Choi SW, O'Reilly PF. PRSice-2: Polygenic Risk Score software for biobank-scale data. *GigaScience*. 2019;8(7):giz082. doi:10.1093/gigascience/giz082
39. Khera AV, Chaffin M, Aragam KG, et al. Genome-wide polygenic scores for common diseases identify individuals with risk equivalent to monogenic mutations. *Nat Genet*. 2018;50(9):1219-1224. doi:10.1038/s41588-018-0183-z
40. Coleman JRI, Peyrot WJ, Purves KL, et al. Genome-wide gene-environment analyses of major depressive disorder and reported lifetime traumatic experiences in UK Biobank. *Mol Psychiatry*. 2020;25(7):1430-1446. doi:10.1038/s41380-019-0546-6
41. McGovern DPB, Taylor KD, Landers C, et al. MAGI2 genetic variation and inflammatory bowel disease. *Inflamm Bowel Dis*. 2009;15(1):75-83. doi:10.1002/ibd.20611
